# Hydrogen Boride Sheets and Copper Nanoparticle Composites as a Visible‐Light‐Sensitive Hydrogen Release System

**DOI:** 10.1002/smll.202404986

**Published:** 2024-09-23

**Authors:** Andi Mauliana, Akira Yamaguchi, Takahiro Kondo, Masahiro Miyauchi

**Affiliations:** ^1^ Department of Materials Science and Engineering School of Materials and Chemical Technology Tokyo Institute of Technology Meguro‐ku Tokyo 152–8552 Japan; ^2^ Department of Materials Science Institute of Pure and Applied Sciences University of Tsukuba Tsukuba 305‐8573 Japan; ^3^ The Advanced Institute for Materials Research Tohoku University 2‐1‐1, Sendai Miyagi 980–8577 Japan; ^4^ Tsukuba Research Center for Energy Materials Science Institute of Pure and Applied Sciences and R&D Center for Zero CO_2_ Emission Functional Materials University of Tsukuba Tsukuba 305‐8573 Japan

**Keywords:** copper nanoparticles, hydrogen boride, hydrogen carrier, 2D material, visible‐light

## Abstract

Hydrogen boride (HB) sheet is a new class of 2D materials comprising hydrogen and boron, synthesized through ion‐exchange and exfoliation techniques. HB sheets can release hydrogen (H_2_) under light irradiation and is predicted to be a promising H_2_ storage material. However, its application is limited to the UV region. One approach to enable a visible‐light‐driven system is the utilization of plasmonic metallic nanoparticles. The present study reports H_2_ release from copper (Cu) nanoparticle‐modified HB sheet (HB/Cu) under visible‐light irradiation. Copper nanoparticles possess unique and strong plasmonic responses in the visible‐light range, making them ideal light absorbers in this system. HB/Cu nanocomposites are synthesized using a simple mixture of copper acetate and HB sheets in acetonitrile, where HB sheets reduced copper ions to metal copper nanoparticles. The photoirradiation results shows that HB/Cu nanocomposites released more H_2_ than the bare HB sheets under visible‐light irradiation. This is probably due to the plasmonic photothermal effect of copper metal, which enhances H_2_ generation from the HB sheets. This material offers a viable and cost‐effective approach for developing visible‐light‐sensitive systems.

## Graphical abstract

This study explores the enhancement of hydrogen release from hydrogen boride (HB) sheets modified with copper nanoparticles (HB/Cu) under visible‐light irradiation. The copper nanoparticles’ plasmonic effects improve H_2_ generation compared to bare HB sheets. The simple synthesis method and effective light absorption make HB/Cu a promising, cost‐effective material for visible‐light‐sensitive hydrogen release systems.

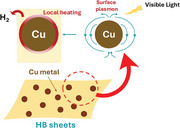

## 1 Introduction

Increasing energy demand and the depletion of fossil fuel reserves are the world's current challenges. Furthermore, the combustion of fossil fuels produces carbon dioxide, causing serious climatic issues. These issues have motivated many researchers to look for alternative resources, minimizing the dependence on fossil‐fuel‐based energy resources.^[^ ^1^ ^]^ Hydrogen (H_2_) is a prospective energy source because of its excellent energy efficiency, environmental benefits (combustion and fuel cell engines do not produce exhaust other than water vapor), and non‐toxic nature.^[^ ^2^, ^3^ ^]^ The most crucial aspect of the development of hydrogen energy is its storage capacity. Unfortunately, safe and compact hydrogen storage is difficult, limiting its extensive application as an energy carrier. Hydrogen is typically compressed or liquefied in tanks by conventional methods.^[^ ^4^ ^]^ The development of materials, such as metal hydrides and carbon structures, has been the focus of several studies to overcome the limitations of conventional hydrogen storage tanks. The only disadvantage of these systems is that they must provide an adequate hydrogen storage capacity or control the adsorption and desorption of hydrogen.^[^ ^5^, ^6^ ^]^ Recently, hydrogen boride (HB) sheet has attracted significant attention as a promising group of 2D materials with potential applications in the future in various fields,^[^ ^7^ ^]^ while boron‐based 2D materials were reported.^[^ ^8^, ^9^, ^10^, ^11^ ^]^ HB sheets consist of boron and hydrogen atoms arranged in a layered structure and exhibit unique properties that make them highly attractive for diverse applications. The material primarily consists of a corrugated boron (B) network adorned with three‐center, two‐electron B─H─B bridging bonds and two‐center, two‐electron B─H terminal bonds.^[^ ^12^ ^]^ The HB sheets exhibit semi‐metallic physical properties and electrical conductivity.^[^ ^12^, ^13^, ^14^ ^]^ Moreover, HB sheets have the potential for hydrogen storage and release owing to the hydrogen atoms, high gravimetric hydrogen capacity (8.5 wt.%), lightweight, and large specific surface area.^[^ ^7^, ^15^, ^16^ ^]^ It has been reported that HB sheets can release their hydrogen by external stimuli, such as heat treatment at a temperature of 150–200 °C^[^ ^7^ ^]^ and electricity.^[^ ^17^ ^]^

In addition to heat treatment and electrolytic H_2_ release, UV irradiation reportedly releases gaseous H_2_ molecules from HB sheets.^[^ ^16^ ^]^ However, HB sheets are not responsive to visible light for H_2_ release. Efficient utilization of the visible‐light sensitivity of HB sheets is crucial for scalable hydrogen production because solar radiation mainly consists of visible light (≈42%), with UV light comprising only a small portion (≈8%).^[^ ^18^ ^]^ Therefore, the development of visible‐light‐driven H_2_ release from HB sheets is essential. In a previous study,^[^ ^19^ ^]^ an approach to enable hydrogen release from HB sheets under visible light involved using the N3 dye, a ruthenium (Ru)‐based dye, as a visible‐light absorber. The hydrogen release occurs because excited electrons are injected from the dye's LUMO band into the conduction band of the HB sheets. However, this system has several limitations: Ru‐based dyes are expensive, and the N3 dye undergoes degradation due to self‐oxidation.

In this study, plasmonic metallic nanoparticles^[^ ^20^ ^]^ were utilized to produce H_2_ from HB sheets under visible‐light irradiation. While gold and silver nanoparticles are commonly employed for this purpose, they are expensive.^[^ ^21^ ^]^ Therefore, we used copper (Cu) nanoparticles as an alternative to gold and silver nanoparticles to induce H_2_ release under visible light in a Cu nanoparticle‐modified HB sheet (HB/Cu). Copper nanoparticles possess unique plasmonic properties, displaying a strong plasmonic response within the visible‐light range, making them ideal light absorbers for this system.^[^ ^22^ ^]^ Moreover, Cu‐metal‐modified HB sheets are a more economical and stable alternative to noble metals. The reduction of Cu ions onto the surface of the HB sheets results in the formation of HB/Cu nanocomposites, showing promising potential for visible‐light‐induced H_2_ release.

## 2 Results and Discussion

### 2.1 Characterization of HB Sheets and HB/Cu Nanocomposite

**Figure 1:**
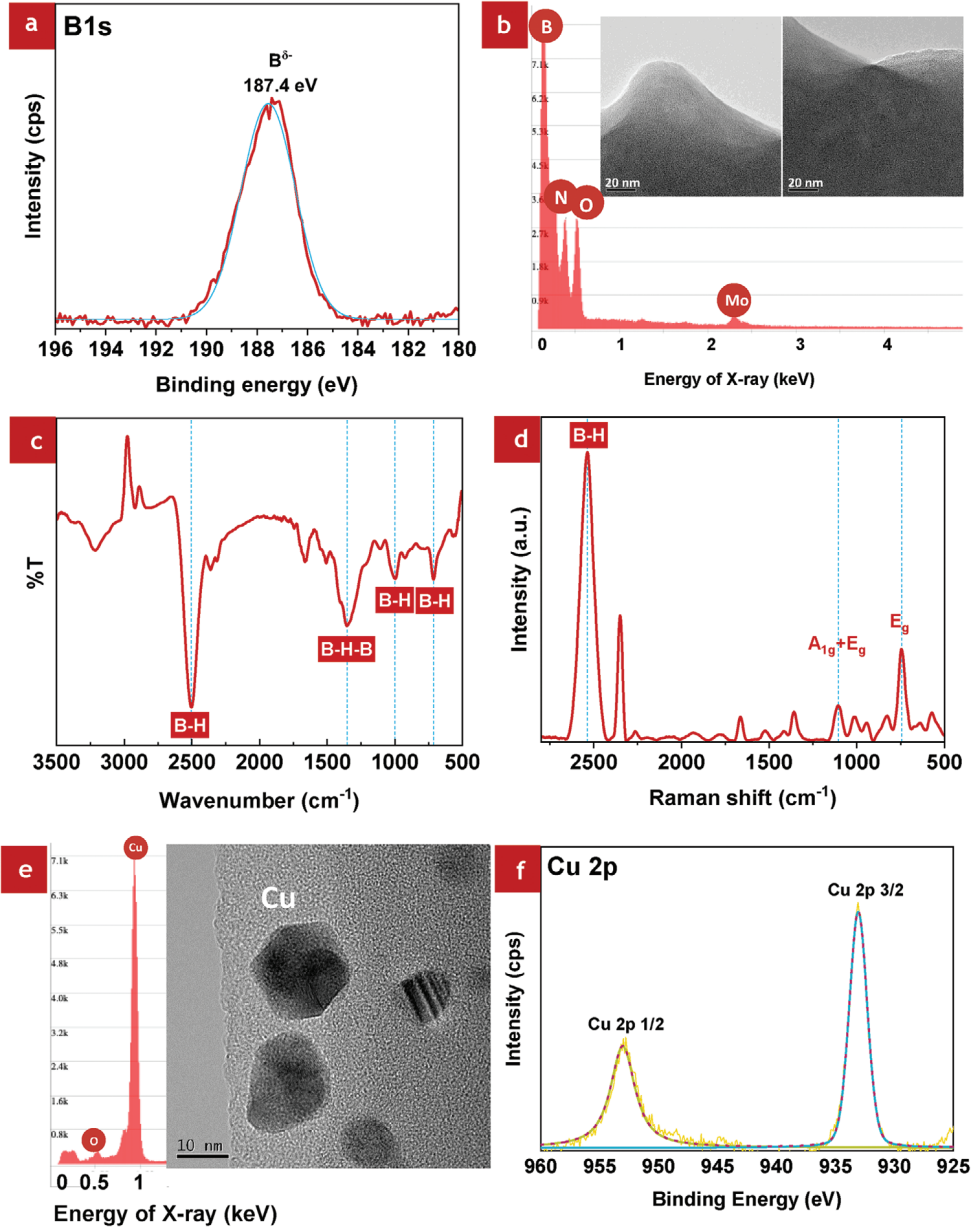
Characterization of bare HB sheets: a) XPS analysis, b) TEM and EDS, c) FT‐IR spectrum, and d) Raman spectrum. Characterization of HB/Cu nanocomposites (with the molar ratio of 100:5): e) TEM and EDS, and f) XPS of Cu‐2p orbital.

HB sheets with an average yield percentage of 61.2% were synthesized. The X‐ray photoelectron spectroscopy (XPS) spectrum of boron 1s orbital (B‐1s) of the bare HB sheets is shown in **Figure** **1**a. The XPS spectrum revealed a peak at 187.4 eV, indicating the presence of negatively charged boron species (*B* ^δ −^). The peak ≈193 eV, corresponding to positively charged oxidized boron species (*B* ^δ +^), was absent in the HB sheets. The high purity of the synthesized HB sample is confirmed from the single peak in the B‐1s spectrum, devoid of signatures of oxidized components.

Figure 1b shows the transmission electron microscopy (TEM) images and the energy‐dispersive X‐ray spectroscopy (EDS) spectrum of bare HB sheets. Powder samples were used to characterize HB sheets using electron microscopy under vacuum. The HB sheets dispersion was vacuum‐dried in a glove box under a nitrogen atmosphere. The TEM images indicate the sheet‐like morphology of the HB sheets. The EDS analysis revealed the presence of boron (B) in HB sheets. The presence of nitrogen (N) and oxygen (O) is likely from the acetonitrile used as the solvent. Furthermore, the molybdenum (Mo) peak in the EDS spectrum is from the microgrid on which the HB sheets were fixed for TEM observation. Fourier transform infrared (FT‐IR) spectrum (Figure 1c) confirmed the B─H bonds, validating the successful synthesis of HB sheets. Peaks corresponding to B─H stretching (2500 cm^−1^), B─H bending (715 and 1000 cm^−1^), and B─H─B stretching (1360 cm^−1^) were observed in the HB nanosheets, which is consistent with the previous reports.^[^ ^7^, ^12^, ^16^, ^23^, ^24^, ^25^ ^]^ The broad absorption peak observed above 3000 cm^−1^ suggests the potential presence of water impurities. It is noteworthy that despite the presence of the water, HB exhibits a low reactivity toward hydrolysis.^[^ ^26^ ^]^ Rojas et al. also reported the absence of detectable boric acid signals in the FT‐IR spectra and the absence of oxidized boron species in the XPS spectra of HB sheets after exposure to water. Figure 1d shows the Raman spectrum of HB sheets. A strong peak observed at 2536 cm^−1^ signifies the stretching vibration of the B─H bonds, indicative of the presence of terminal hydrogen atoms in HB sheets. Another prominent peak observed at 743 cm^−1^ corresponds to the vibrations of the B─B bonds (E_g_ mode). The E_g_ mode of the B─B bonds is formed by a combination of two vibration modes (ω_1_ and ω_2_). Vibration mode, ω_1_, is caused by the simultaneous compression of six external two‐center bonds of the icosahedron whereas ω_2_ involves the compression of two bonds, the stretching of two bonds, and the absence of strain in two bonds. Furthermore, a peak at 1100 cm^−1^, corresponding to A_1g_ and E_g_ modes, can be linked to either a rocking mode of B‐H or a B‐H deformation.^[^ ^14^, ^27^, ^28^, ^29^, ^30^ ^]^

Figure 1e shows the TEM image and EDS result of the HB/Cu nanocomposite with a molar ratio of 100:5, which exhibited the highest H_2_ production property, as mentioned later. The results indicate the distribution of Cu nanoparticles of an average size of 6.6 nm (Figure S2, Supporting Information), visible as black spots on the HB sheet layer. The EDS results also confirm Cu nanoparticles from the Cu peak in the graph (Figure 1e). In Figure S2a (Supporting Information), the appearance of the B peak indicates the presence of HB sheets. The N and O peaks are from the acetonitrile used during the preparation of the powder by vacuum evaporation of the HB/Cu solution. EDS analysis confirmed the formation of HB/Cu nanocomposites.

Figure 1f shows the XPS measurements to analyze further the elemental compositions and chemical states of the HB/Cu nanocomposites. The B‐1s peak (187.8 eV) in the XPS spectrum, indicating negatively charged boron (boride), corresponds to the HB sheets (Figure S3, Supporting Information). Moreover, this HB/Cu nanocomposite material shows a small peak corresponding to oxidized boron (B^δ+^) at 192.5 eV after adding the Cu ions into the HB solution. Additionally, in Figure 1f, the two core‐level peaks of Cu‐2p in the range of 925–960 eV were assigned to metallic Cu states, as reported in previous studies.^[^ ^31^, ^32^ ^]^ The peaks at binding energies 933  and 953 eV correspond to Cu‐2p (3/2) and Cu‐2p (1/2), respectively, confirming that the black spots in the TEM images are Cu nanoparticles. The reduction of Cu ions to Cu nanoparticles occurs via electron donation from the HB sheets, resulting in the partial oxidation of boron. In addition, the FT‐IR spectra of HB/Cu nanoparticles with ratios of 100:1, 100:5, and 100:10 are shown in the Supporting Information (Figure S4, Supporting Information). The results confirm the presence of H─B bonding at wavenumber ≈2500 cm^−1^ for all HB/Cu samples, regardless of their ratio, indicating that the H─B bonding was preserved across all compositions.

**Figure 2:**
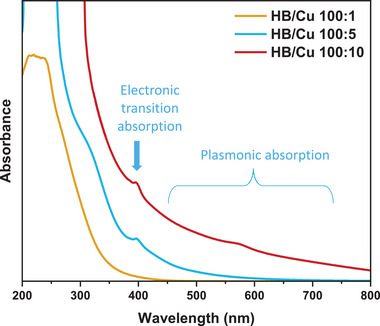
UV–vis spectra of HB/Cu nanocomposites of 100:1, 100:5, and 100:10.

UV–vis spectroscopy measurements were conducted to examine the optical properties of the HB/Cu nanocomposites. **Figure** **2** shows the UV–vis spectra of HB/Cu nanocomposites with molar ratios of 100:1, 100:5, and 100:10. The results indicated a change in the absorption toward longer wavelengths as the concentration of Cu in the HB/Cu nanocomposite solution increased. The observed shift in these UV–vis spectra is likely due to the plasmon resonance of the Cu nanoparticles on the HB sheets.^[^ ^33^ ^]^ The surface plasmon resonance effect on the absorption shift is probably due to the increased metal nanoparticle size. This correlation is supported by several studies, indicating a red shift in the absorption peak as the metal nanocluster particle size increases. This red shift in the absorption peak may be caused by surface plasmon coupling arising from the proximity of the Cu metal nanoparticles and a decrease in the distance between them.^[^ ^34^ ^]^ Figure S5, Supporting Information presents the UV–vis results of the bare HB sheets and free Cu(CH_3_COO)_2_ solution and compares them with those of the HB/Cu nanocomposites. The spectrum of the Cu(CH_3_COO)_2_ solution exhibits a strong peak at a wavelength of ≈670 nm, which gives the solution a blue color because the Cu^2+^ ion is absorbed in that wavelength range. The absorption at ≈650 nm became negligible after mixing the Cu^2+^ ions with the HB sheets, indicating the reduction of Cu^2+^ ions to Cu metal by the HB sheets. As shown in Figure 2, two types of absorption occur in the visible‐light region, i.e., plasmonic absorption of Cu metal over 450 nm and a small absorption peak at 400 nm, especially in the HB/Cu nanocomposites with ratios of 100:5 and 100:10. According to previous studies, the peak at ≈400 nm can be attributed to the interband absorption of Cu nanoparticles from the d‐band to the sp‐band.^[^ ^22^, ^34^, ^35^ ^]^

### 2.2 Hydrogen Release from HB/Cu under Visible‐Light Irradiation

**Figure 3:**
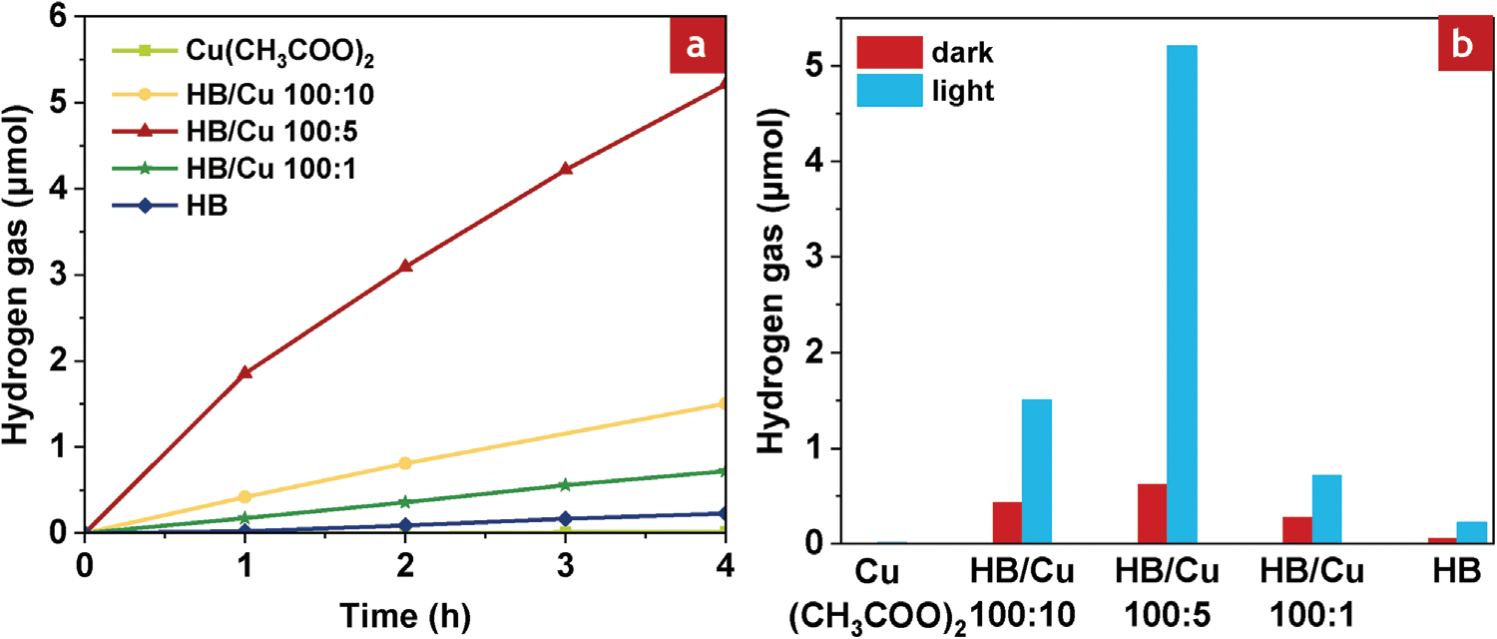
Hydrogen release from HB/Cu nanocomposites a) under light irradiation and b) amount of generated hydrogen amount after 4 h.

The H_2_ release properties of the HB/Cu nanocomposites were evaluated in a liquid medium using a closed reactor filled with an inert gas. The liquid medium acts as a coolant to keep the liquid temperature under light irradiation at 298 K, close to room temperature. An investigation was also conducted under dark conditions to observe the effect of the photoinduced H_2_ release from the HB/Cu nanocomposites. **Figure** **3** shows the H_2_ release properties of the HB sheets, HB/Cu nanocomposites with ratios of 100:1, 100:5, and 100:10, and Cu(CH_3_COO)_2_ in acetonitrile under visible‐light irradiation for 4 h. The HB/Cu nanocomposites exhibited significant H_2_ release under visible‐light irradiation compared to bare HB sheets without Cu. The bare HB sheets exhibited only a small amount of H_2_ gas because of their inability to absorb visible‐light.^[^ ^16^ ^]^ In the case of Cu(CH_3_COO)_2_ in acetonitrile, no H_2_ gas was observed, indicating that free Cu^2+^ ions cannot enhance the H_2_ released in the HB/Cu nanocomposites. Among the HB/Cu nanocomposites with different ratios, HB/Cu with a ratio of 100:5 showed remarkable performance compared to the nanocomposites with ratios of 100:1 and 100:10. The small amount of Cu in HB/Cu 100:1 did not have a significant effect on H_2_ release, possibly because of negligible plasmonic effect in the system. As shown in the UV–vis spectrum (Figure 2), the visible‐light absorption of HB/Cu 100:1 was limited, but it still showed a higher H_2_ release than the bare HB sheets. In contrast, HB/Cu with a ratio of 100:10 produced more H_2_ than HB/Cu 100:1, but less H_2_ than HB/Cu 100:5. This is probably because the higher amounts of Cu in the HB/Cu nanocomposite (100:10) can reduce the H_2_ release property of the nanocomposite owing to the aggregation of the metal, leading to a reduction in the active surface area and limited plasmonic effect. The evaluation under light and dark conditions evidence that H_2_ release from the HB/Cu nanocomposites is stimulated by visible‐light irradiation. The visible‐light sensitivity of these nanocomposites suggests the plasmon resonance effect of metallic Cu. Several studies have also demonstrated improvements in the performance of photocatalysts using metallic Cu with plasmonic properties.^[^ ^36^, ^37^, ^38^, ^39^ ^]^

Further, we examined the light intensity dependence on the H_2_ release from HB/Cu nanoparticles by varying the distance between the light source and the sample to 16, 12, and 8 cm. The results are shown in the Supporting Information (Figure S6, Supporting Information). The findings indicate that higher light intensity leads to increased hydrogen production, suggesting that the process is directly influenced by the incident light intensity due to the availability of more photons providing the energy required for hydrogen release.

**Figure 4:**
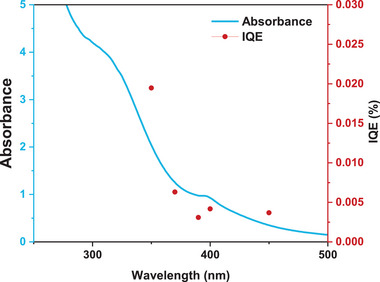
Action spectrum of HB/Cu 100:5 nanocomposite irradiated under visible‐light source for 30 min measurement (red circles). The blue line shows the absorbance of HB/Cu 100:5 nanocomposite.

**Figure** **4** shows the action and absorption spectra of the HB/Cu 100:5 nanocomposite, evaluated using a 500 W xenon lamp with specific bandpass filters. The action spectrum reveals that the internal quantum efficiency (IQE) for each wavelength closely corresponds to the absorption spectrum, indicating that the plasmonic resonance in the Cu metal distributed on the surface of the HB sheets has a predominant influence on the H_2_ release in the HB/Cu nanocomposite. This action spectrum confirms the importance of the plasmonic effect in improving the H_2_ release of the HB/Cu nanocomposites.

**Figure 5:**
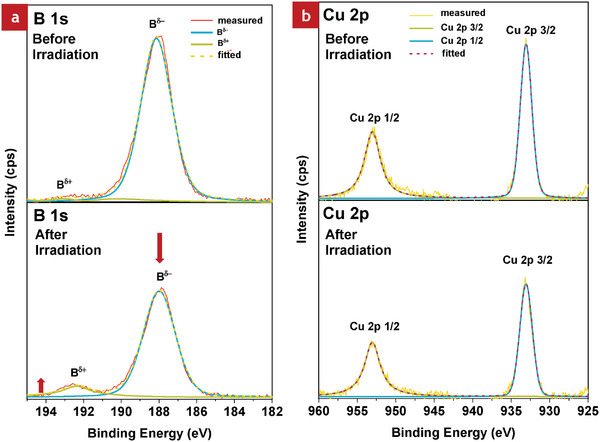
XPS spectra of HB/Cu before (top) and after irradiation (bottom) with core‐level spectra of a) B‐1s and b) Cu‐2p. The molar ratio of the HB/Cu composite was 100:5.

**Figure** **5** shows the XPS results of the HB/Cu nanocomposites before and after light irradiation. The data revealed significant changes in the chemical states of the samples. The B‐1s peak of the positively charged B^δ+^ species at 192.5 eV increased, while that of B^δ−^ species at 187.8 eV decreased after visible‐light irradiation. These results indicate that oxidation occurred during H_2_ release under visible‐light irradiation. A similar trend was reported for the heat treatment process, where B^δ+^ species increased after H_2_ release at high temperature.^[^ ^16^ ^]^ Therefore, in the present study, the localized heating effect from plasmon absorption in the Cu nanoparticles led to H_2_ generation under visible‐light. Furthermore, in the Cu‐2p peaks, no chemical shift was observed after light irradiation, indicating that Cu remained in its metallic state even after light irradiation. The presence of metallic Cu further supports the role of the plasmonic Cu metal in enhancing the H_2_ release properties of the HB/Cu nanocomposites.

Additionally, FT‐IR measurements were performed to observe the differences in the B─H bonds of the HB/Cu nanocomposites before and after visible‐light irradiation, as illustrated in the supporting information (Figure S7, Supporting Information). The peak at ≈2500 cm^−1^, indicating the B─H bonds in the HB sheets, decreased after visible‐light irradiation. The observed reduction in the peak related to B─H bonds indicated the release of H_2_ within the system. Similar results have been reported for UV‐induced^[^ ^16^ ^]^ and electrolytic H_2_ releases.^[^ ^17^ ^]^

### 2.3 Mechanism of Hydrogen Release

**Figure 6:**
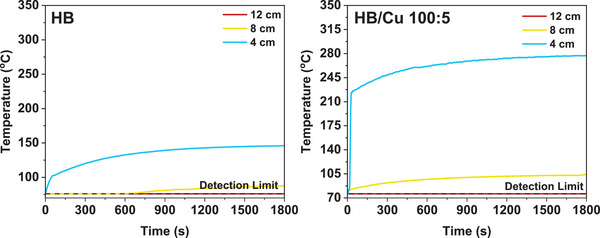
Surface temperature under visible light irradiation with different intensities by varying the distances from the light source to samples (4, 8, 12 cm). The left panel shows the results of bare HB, and the right panel shows those of HB/Cu with a ratio of 100:5.

To confirm the effect of Cu nanoparticle in the HB/Cu nanocomposites and to elucidate the H_2_ release mechanism, we measured the surface temperature in argon atmosphere under visible light irradiation using a radiation thermometer (Figure S8a, Supporting Information) equipped with a UV cutoff filter. We analyzed the relationship between temperature changes over the time at varying the distances from the sample to light source (4, 8, and 12 cm) with a detection limit of 76 °C for bare HB and HB/Cu 100:5 samples (**Figure** **6**). During the initial few seconds, the light source was off, and after it was turned on, the temperature gradually increased. The initial temperature recorded was 76 °C, which is at the detection limit, indicating that the actual temperature was likely below 76 °C. The results show that the temperature of the HB/Cu 100:5 sample increased more significantly over time compared to bare HB, suggesting a local heating effect due to the presence of Cu nanoparticle. As shown in Figure S8b, Supporting Information, as the light intensity increased and the distance to the light source decreased, the temperature of the HB/Cu nanocomposites rose more significantly compared to bare HB. After 30 min of irradiation at high light intensity, the maximum temperature of bare HB reached 146 °C, whereas the temperature of the HB/Cu 100:5 climbed to 280.5 °C. This substantial difference indicates the presence of Cu nanoparticles enhances the photothermal effect, leading to higher temperatures. Although the surface temperature was measured under argon gas, the local heat would be generated around Cu nanoparticles even in solution media. The elevated temperature in the HB/Cu nanocomposite boosts H_2_ release, with higher light intensity accelerating the process and generating more hydrogen.

**Figure 7:**
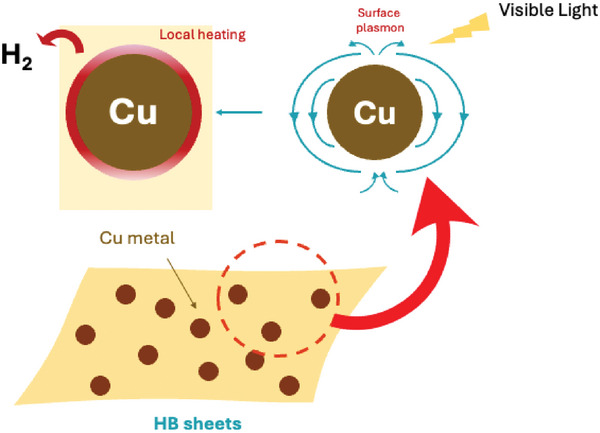
Expected mechanism of H_2_ release from HB/Cu nanocomposites under visible‐light irradiation.

**Figure** **7** illustrates the expected mechanism of visible‐light‐induced H_2_ release from the HB/Cu nanocomposite system. Based on the action spectrum shown in Figure 4, though the contribution of the interband transition at 400 nm was not very high, the H_2_ release on irradiation with 450 nm light was significant. These results strongly imply that plasmon absorption in Cu metal is the dominant process for H_2_ release from the HB/Cu nanocomposites under visible‐light irradiation. There are two types of plasmonic effects, i.e., hot carrier generation and photothermal effect. When Cu metal absorbs visible‐light, the incident light excites surface plasmons on the metal surface, leading to excited electrons in Cu metal. These electrons are possibly transferred from the Cu nanoparticles to the conduction band of the HB sheets, resulting in H_2_ release by the self‐reduction of the HB sheets. However, the XPS results shown in Figure 5a demonstrate the formation of the oxidized species. Based on these results, the photothermal effect is more plausible than the effect of excited electrons in Cu nanoparticles under visible‐light irradiation. In a previous study, oxidized boron species were observed in the XPS spectrum after H_2_ release via heat treatment,^[^ ^16^ ^]^ similar to the results shown in Figure 5a. Additionally, the results of surface temperature (Figure 6) suggest this effect. These results suggest that the localized heating effect from plasmon absorption contributes to the H_2_ release from the HB sheets. The enhanced H_2_ release of the HB/Cu nanocomposite in the visible‐light range was predominantly due to the photothermal effect of plasmon absorption in the Cu nanoparticles. This finding further reinforces the significance of the plasmonic effect and localized heating in facilitating H_2_ release in the nanocomposite system.^[^ ^40^, ^41^, ^42^, ^43^ ^]^

## 3 Conclusion

In the study, HB/Cu nanocomposites were successfully synthesized by exploiting the reduction properties of HB sheets. The HB/Cu nanocomposite enabled the release of H_2_ from HB under visible‐light irradiation. The action and the UV spectra provide strong evidence supporting the light absorption and plasmon effects in Cu metal in this system, enhancing the hydrogen generation of HB nanosheets under visible‐light irradiation. As a result, a visible‐ light‐sensitive system can be developed using earth‐abundant and cost‐effective plasmonic Cu metal.

## 4 Experimental Section

### Synthesis of HB Sheets

Magnesium diboride (MgB_2_) powder (99% trace metal basis, Sigma–Aldrich Co., St. Louis, MO, USA), ion‐exchange resin (Amberlite IR120B hydrogen form, Organo Corp., Tokyo, Japan), and acetonitrile (super‐dehydrated, 99.8%, FUJIFILM Wako Pure Chemical Co., Ltd.) were used.

0.5 g MgB_2_ powder and 30 mL ion‐exchange resin (prewashed with acetonitrile) were introduced into a 200 mL acetonitrile in a flask. This procedure was conducted inside a glove box in a nitrogen environment at room temperature, and the mixture was stirred at 400 rpm for 2 days. Precipitates containing unreacted MgB_2_ and ion‐exchange resin were removed by filtration, employing a 0.2 µm pore filter made of PTFE membrane (Omnipore Membrane Filters, Merck Millipore, Billerica, MA). The resulting filtrate was transferred to a cooling machine at ≈233 K and kept there for 45 min to precipitate the boric acid impurity. The filtrate underwent two additional filtration processes to remove the impurities. Subsequently, the filtrate was dried in a vacuum, resulting in the formation of a yellow powder of HB sheets.

### Preparation of HB/Cu Nanocomposites

HB nanocomposites modified with Cu metal nanoparticles were prepared by taking advantage of the reduction properties of HB sheets, according to Ito et al.^[^ ^32^ ^]^ To prepare the HB/Cu nanocomposite, Cu(II) acetate (Cu(CH_3_COO)_2_), 98%, Aldrich) the powder was dissolved in acetonitrile (super‐dehydrated 500 mL, 99.8%, FUJIFILM Wako Pure Chemical Co., Ltd.) at 0.0005, 0.0025, and 0.005 mol L^−1^. The HB sheet powder was simultaneously added to another bottle of acetonitrile to achieve a concentration of 0.05 mol L^−1^. Subsequently, the Cu(II) acetate and HB solutions were combined at a 1:1 volume ratio to produce HB/Cu nanocomposites in 100:1, 100:5, and 100:10 proportions. The solution was dried via vacuum evaporation to obtain powdered HB/Cu nanocomposites for characterization. All experiments were conducted in a glove box under a nitrogen atmosphere.

### Characterization

The characterization of HB/Cu involves various analytical techniques. Transmission electron microscopy (TEM), scanning transmission electron spectroscopy (STEM), and energy‐dispersive X‐ray spectroscopy (EDS) were performed using a JEM‐2100F TEM/STEM instrument (JEOL Ltd., Jiapan) operating at an electron beam acceleration voltage of 200 kV. X‐ray photoelectron spectroscopy (XPS) measurements were carried out using a JPS 9010 TR instrument (JEOL, Ltd., Japan) equipped with an Mg Kα X‐ray source (1486.6 eV). The samples for XPS analysis were attached to a carbon tape, and the XPS peaks were calibrated using the carbon 1s orbital peak. Fourier transform infrared (FT‐IR) spectra were recorded using an FT/IR‐6100 instrument (JASCO, Co., Ltd., Japan) with an attenuated total reflection (ATR) unit. For the FT‐IR analysis, samples were drop‐cast and adhered to the sample holder in the ATR unit. The optical absorbance of the HB/Cu nanocomposite solution was measured using an UV–vis spectrometer (JASCO, V‐660).

### Hydrogen Release Property

The experimental setup for the H_2_ release measurement is shown in the (Figure S1a, Supporting Information). Each HB/Cu solution (5 mL) was added into a closed quartz reactor filled with nitrogen gas in its headspace. The measurements were conducted under dark and light conditions at a distance of 8 cm from the light source, and the solution was stirred at a speed of 250 rpm. The visible‐light source was a 500 xenon (Xe) lamp with a UV cutoff filter which can cut off wavelengths below 400 nm. The spectra of the light source with and without the UV cutoff filter are shown in the (Figure S1b, Supporting Information). To investigate the action spectrum, bandpass filters of 550, 450, 400, 390, and 350 nm were inserted between the Xe lamp light source and the sample. The amount of H_2_ gas produced was measured using a gas chromatograph (GC) equipped with a thermal conductivity detector (TCD) (SHIMADZU GC‐2014).

The quantum efficiency (*QE*) was calculated using the following equation, considering a two‐electron process for H_2_ generation:

(1) $$\begin{array}{ccc} QE & = & \frac{Rate\;of\;used\;electron\;number\;\left[s^{-1}\right]}{Absorbed\;photon\;number\;\left[s^{-1}\right]}\\ & = & \;\;\frac{H_{2}\;generation\;\left[mol\;s^{-1}\right]\times 2\times N_{A}\left[mol^{-1}\right]}{\sum_{\lambda \;=\;200}^{\lambda \;=\;800}\left\{Absorption\times Incident\;Photon\right\}}\\ & = & \frac{H_{2}\;generation\;\left[mol\;s^{-1}\right]\times 2\times N_{A}\left[mol^{-1}\right]}{\sum_{\lambda \;=\;200\;nm}^{\lambda \;=\;800\;nm}\left\{\left(Absorption\right)\times \frac{\left(E\left[\mu W\cdot cm^{-2}\right]\times 10^{-6}\right)}{h\left[j\cdot s\right]\times c\left[m\cdot s^{-1}\right]/\left(\lambda \left[nm\right]\times 10^{-9}\right)}\right\}\times A\left[cm^{2}\right]} \end{array}$$

(2) $$Absorption=1-Transmittance\;\left(recorded\;by\;an\;UV-Vis\;spectroscopy\right)$$

(3) $$N_{A}\left[mol^{-1}\right]\;:\mathrm{Avogadro}\;\mathrm{number}\;\left(6.02\times 10^{23}\right)$$

(4) $$\begin{array}{ccc} & & E\left[\mu W\cdot cm^{-2}\right]\;:\;\mathrm{Light}\;\mathrm{energy}\;\mathrm{when}\;\mathrm{the}\;\mathrm{wavelength}\;\mathrm{is}\;\lambda \;\mathrm{nm}\;\\ & & \;\times \left(\mathrm{recorded}\;\mathrm{by}\;a\;\mathrm{spectroradiometer}\right) \end{array}$$

(5) $$h\left[j\cdot s\right]\;:{\mathrm{Planck}}^{\prime }s\;\mathrm{constant}\left(\;=\;6.62\times 10^{-34}\right)$$

(6) $$c\left[m\cdot s^{-1}\right]\;:\mathrm{Speed}\;\mathrm{of}\;\mathrm{light}\;\left(\;=\;3.0\times 10^{8}\right)$$

(7) $$\lambda \left[nm\right]\;:\;\mathrm{Wavelength}$$

(8) $$A\left[cm^{2}\right]\;:Area\;\mathrm{irradiated}\;\mathrm{by}\;\mathrm{light}$$

## Conflict of Interest

The authors declare no conflict of interest.

## Supporting information



Supporting Information

## Data Availability

The data that support the findings of this study are available from the corresponding author upon reasonable request.
