# Supplementary material for: Hydrogen Boride Sheets and Copper Nanoparticle Composites as a Visible‐Light‐Sensitive Hydrogen Release System
Source: Small. 2024 Sep 23;20(49):2404986. doi: 10.1002/smll.202404986 (PMC11618728; doi:10.1002/smll.202404986)
Supplement: Supplementary file 1 — Supporting Information [file SMLL-20-2404986-s001.pdf]

NANO · MICRO small

Supporting Information

for *Small*, DOI 10.1002/smll.202404986

Hydrogen Boride Sheets and Copper Nanoparticle Composites as a Visible-Light-Sensitive Hydrogen Release System

Andi Mauliana, Akira Yamaguchi, Takahiro Kondo and Masahiro Miyauchi**

Supporting Information

Hydrogen Boride Sheets and Copper Nanoparticle Composites as a Visible-Light-Sensitive Hydrogen Release System

Andi Mauliana[a], Akira Yamaguchi[a], Takahiro Kondo*[b], Masahiro Miyauchi*[a]

[a] A. Mauliana, A. Yamaguchi, M. Miyauchi
Department of Materials Science and Engineering, School of Materials and Chemical Technology, Tokyo Institute of Technology, Meguro-ku, Tokyo, 152-8552, Japan.
E-mail: mmiyauchi@ceram.titech.ac.jp (M. Miyauchi)

[b] T. Kondo
Department of Materials Science, Institute of Pure and Applied Sciences, University of Tsukuba, Tsukuba 305-8573, Japan.
and
The Advanced Institute for Materials Research, Tohoku University, 2-1-1 Sendai, Miyagi 980-8577, Japan.
and
Tsukuba Research Center for Energy Materials Science, Institute of Pure and Applied Sciences and R&D Center for Zero CO_2 Emission Functional Materials, University of Tsukuba, Tsukuba 305-8573, Japan.
E-mail: takahiro@ims.tsukuba.ac.jp

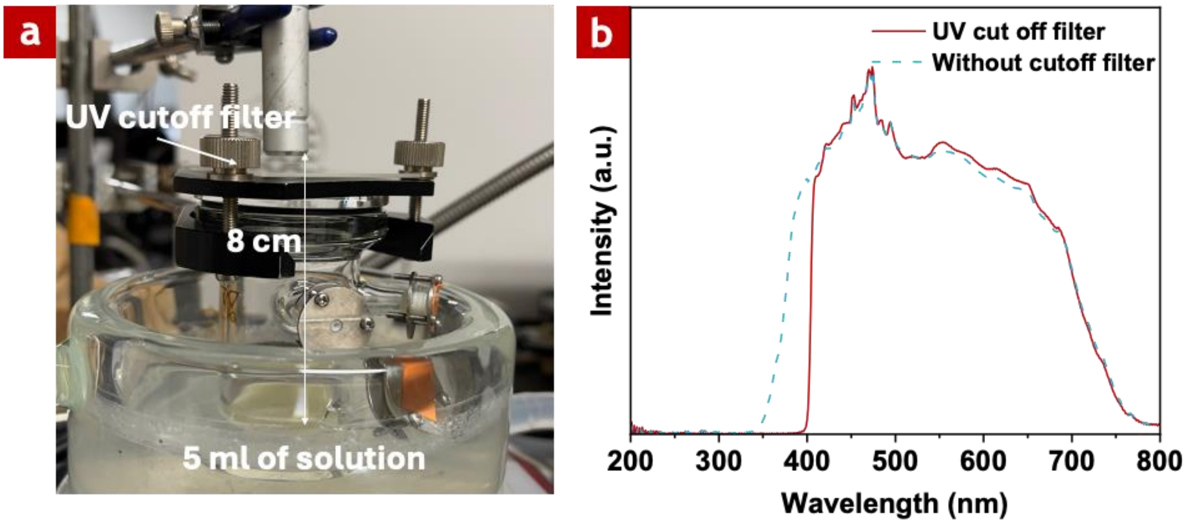

Figure S1. a) The experimental setup for hydrogen release evaluation under visible light irradiation equipped with the UV-cutoff filter, **b)** The spectra of a 500-Xenon lamp as visible light source with (red line) and without UV cutoff filter (blue dash line)

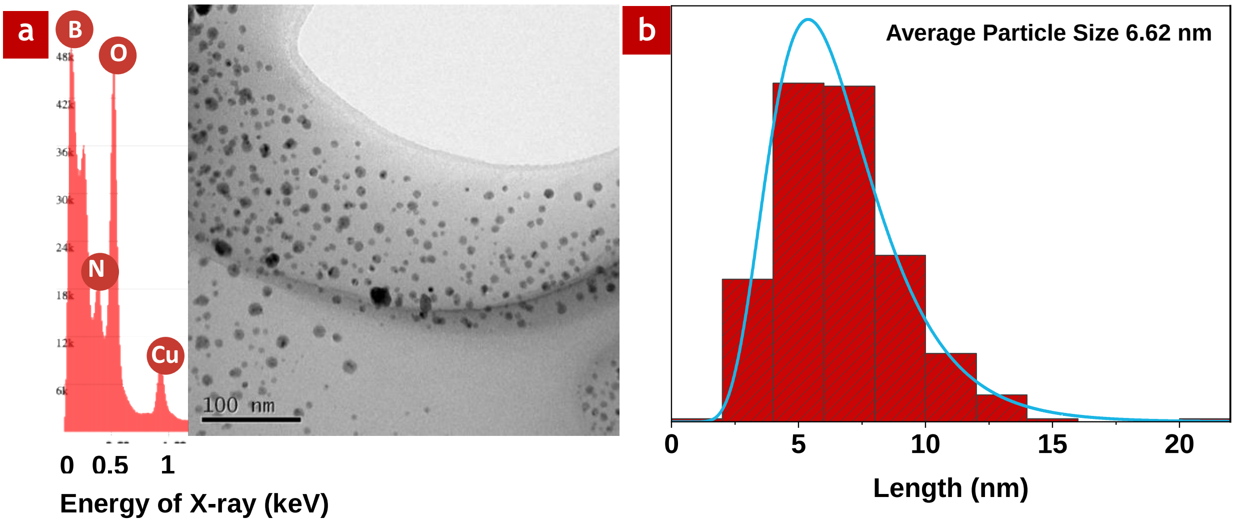

Figure S2. a) TEM-EDS results of HB/Cu nanocomposite (with the molar ratio of 100:5), **b)** Average size distribution of copper metal nanoparticles on HB surface (HB/Cu 100:5)

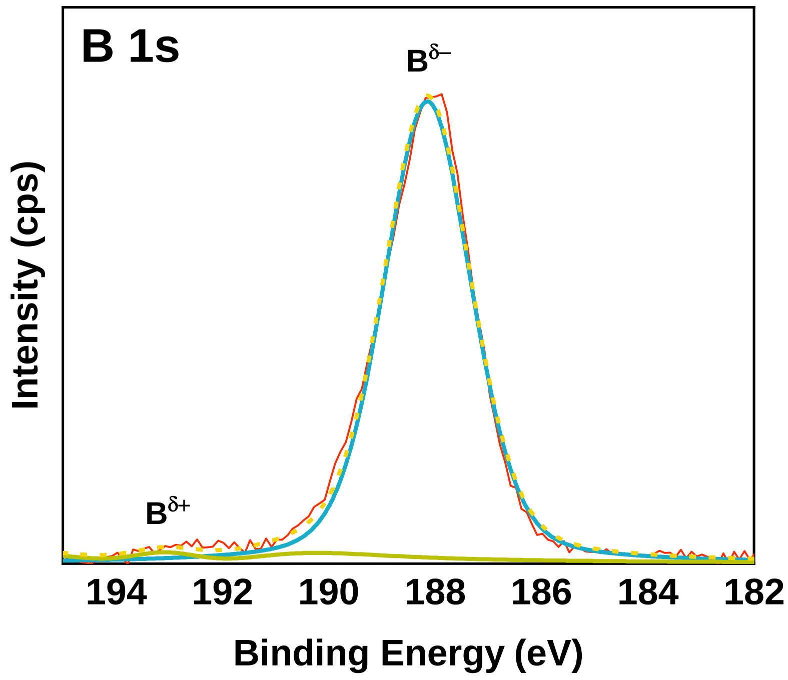

Figure S3. XPS spectra of HB/Cu (with the molar ratio of 100:5) for B-1s orbital.

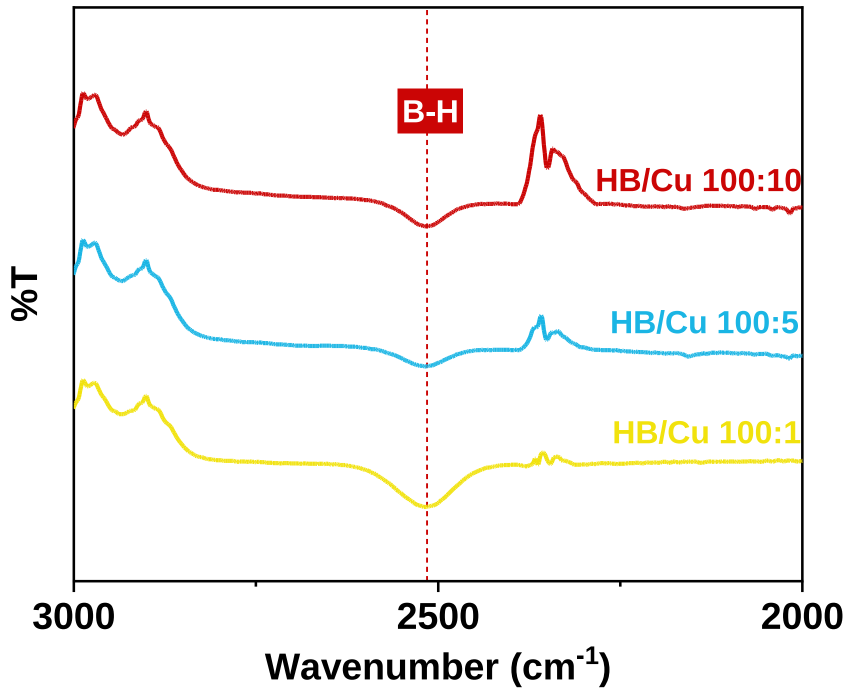

Figure S4. FT-IR spectra of HB/Cu 100:1, HB/Cu 100:5, and HB/Cu 100:10, respectively.

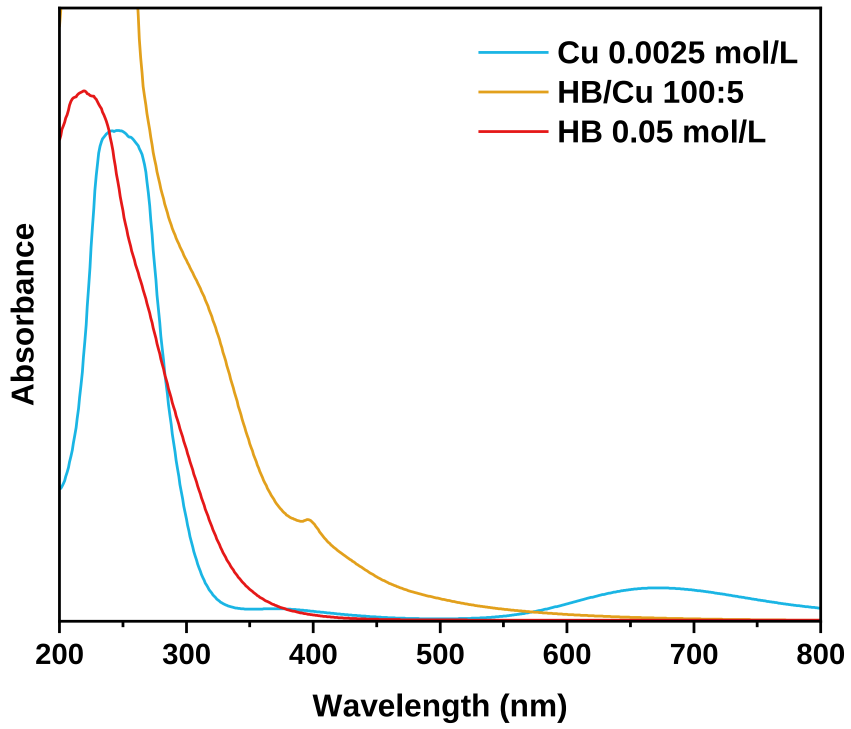

Figure S5. UV-Vis spectra of $Cu(CH_3COO)_2$ in acetonitrile (Cu 0.0025 mol/L), HB/Cu 100:5, and HB in acetonitrile (HB 0.05 mol/L), respectively.

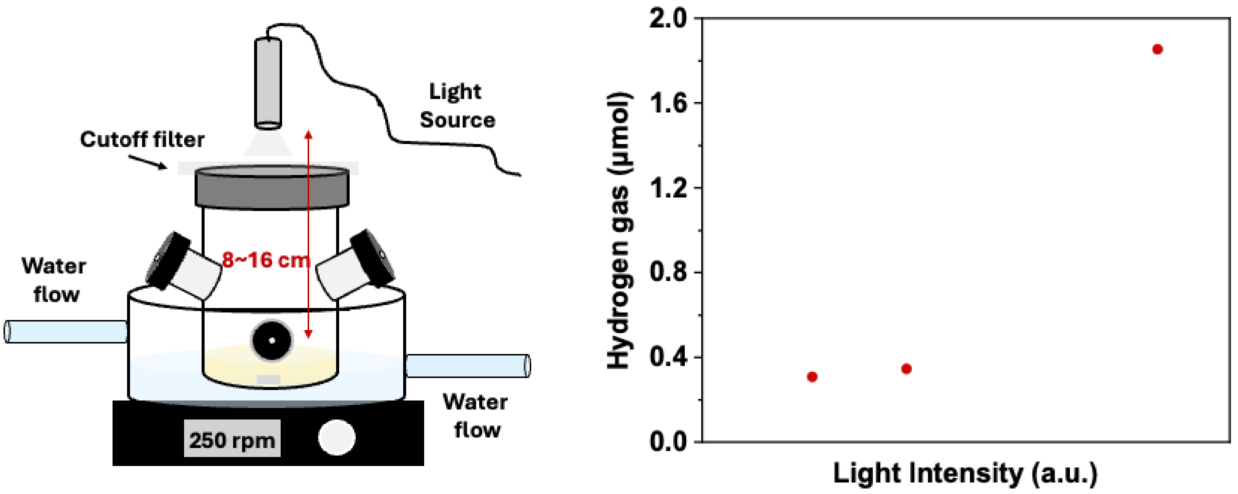

Figure S6. Hydrogen release from HB/Cu 100:5 as a function of light intensity, adjusted by varying the distance between the light source and the sample (16, 12, 8 cm).

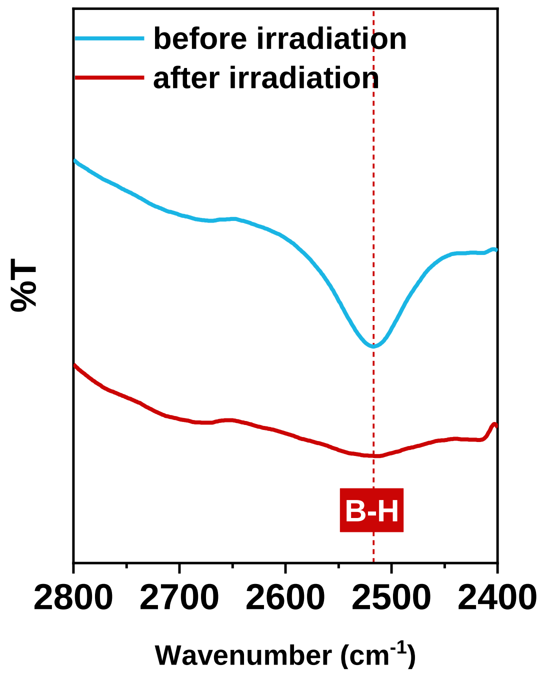

Figure S7. FTIR spectra of HB/Cu before (blue line) and after (red line) irradiation. The molar ratio of the HB/Cu composite was 100:5.

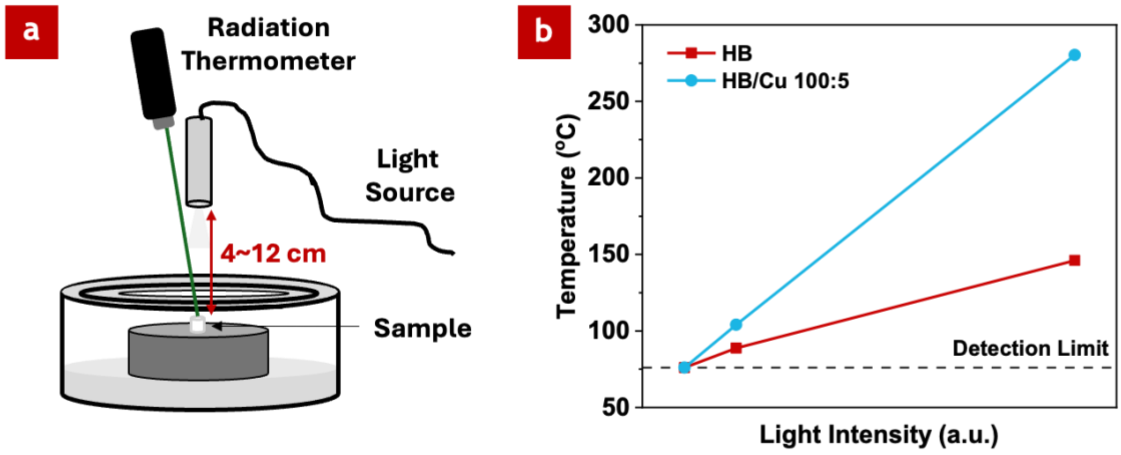

Figure S8. a) Schematic illustration of surface temperature measurement, **b)** light intensity dependence on the temperature change of bare HB sheets and HB/Cu 100:5.
